# Physicochemical and Antioxidant Properties of Acid- and Pepsin-Soluble Collagens from the Scales of Miiuy Croaker (*Miichthys Miiuy*)

**DOI:** 10.3390/md16100394

**Published:** 2018-10-20

**Authors:** Long-Yan Li, Yu-Qin Zhao, Yu He, Chang-Feng Chi, Bin Wang

**Affiliations:** 1 Zhejiang Provincial Engineering Technology Research Center of Marine Biomedical Products, School of Food and Pharmacy, Zhejiang Ocean University, Zhoushan 316022, China; 15576494647@163.com (L.-Y.L.); zhaoy@hotmail.com (Y.-Q.Z.); heyu19950618@163.com (Y.H.); 2 National and Provincial Joint Laboratory of Exploration and Utilization of Marine Aquatic Genetic Resources, National Engineering Research Center of Marine Facilities Aquaculture, School of Marine Science and Technology, Zhejiang Ocean University, Zhoushan 316022, China

**Keywords:** miiuy croaker (*Miichthys miiuy*), scale, acid-soluble collagen (ASC), pepsin-soluble collagen (PSC), antioxidant activity, radical scavenging activity

## Abstract

In this report, acid-soluble collagen (ASC-MC) and pepsin-soluble collagen (PSC-MC) were extracted from the scales of miiuy croaker (*Miichthys miiuy*) with yields of 0.64 ± 0.07% and 3.87 ± 0.15% of dry weight basis, respectively. ASC-MC and PSC-MC had glycine as the major amino acid with the contents of 341.8 ± 4.2 and 344.5 ± 3.2 residues/1000 residues, respectively. ASC-MC and PSC-MC had lower denaturation temperatures (32.2 °C and 29.0 °C for ASC-MC and PSC-MC, respectively) compared to mammalian collagen due to their low imino acid content (197.6 and 195.2 residues/1000 residues for ASC-MC and PSC-MC, respectively). ASC-MC and PSC-MC were mainly composed of type I collagen on the literatures and results of amino acid composition, SDS-PAGE pattern, ultraviolet (UV) and Fourier-transform infrared spectroscopy (FTIR) spectra. The maximum solubility of ASC-MC and PSC-MC was appeared at pH 1–3 and a sharp decrease in solubility was observed when the NaCl concentration was above 2%. Zeta potential studies indicated that ASC-MC and PSC-MC exhibited a net zero charge at pH 6.66 and 6.81, respectively. Furthermore, the scavenging capabilities on 1,1-diphenyl-2-picrylhydrazyl (DPPH) radical, hydroxyl radical, superoxide anion radical and 2,2′-azino-bis-3-ethylbenzothiazoline-6-sulfonic acid (ABTS) radical of ASC-MC and PSC-MC were positively correlated with their tested concentration ranged from 0 to 5 mg/mL and PSC-MC showed significantly higher activity than that of ASC-MC at most tested concentrations (*p* < 0.05). In addition, the scavenging capability of PSC-MC on hydroxyl radical and superoxide anion radical was higher than those of DPPH radical and ABTS radical, which suggested that ASC-SC and PSC-SC might be served as hydroxyl radical and superoxide anion radical scavenger in cosmeceutical products for protecting skins from photoaging and ultraviolet damage.

## 1. Introduction

Collagen is the most abundant protein constituting nearly 30% of all proteins in the animal body and is a primary component of the extracellular matrix [1]. Collagen plays an important role in the formation of organs and maintenance of the structural integrity of cells [2]. Up to the present, genetically distinct 29 types of collagen (type I-XXIX) with right-handed triple helical conformation have been isolated from animal tissue that differ considerably in their amino acid composition, sequence, structural and functional properties [3,4]. Traditionally, collagens were mainly prepared from bovine tendon and porcine skins and have been extensively utilized as biomedical materials for functional food, cosmetics and tissue engineering because of their favorable biological features, such as excellent biodegradability, biocompatibility and weak antigenicity [5,6]. At present, some consumers have paid close attention to the safety of mammalian collagens because of the outbreaks of bovine spongiform encephalopathy, foot mouth disease and other prions disease. In addition, use of mammalian collagen is a hurdle in the development of kosher and halal products due to some religious factors [3,7]. Therefore, the enormous demand for collagen from alternative resources such as aquatic byproducts (skin, bone, swim bladder, scale and fins) has increased for many years due to no dietary restriction and risk of disease transmission [3,8]. Furthermore, effective use of aquatic byproducts to produce high value-added products is an important way to increase the income to the fish processor and protect the environment [9].

An imbalance in pro-oxidant/antioxidant can cause oxidative stress, which further trigger the accumulated reactive oxygen species (ROS) production and result in cell damage and many health disorders, such as skin damage, diabetes mellitus, cancer and inflammatory diseases [10,11]. Therefore, researchers have continued to show an interest in screening naturally-derived antioxidants including collagens and their peptides. Acid-soluble collagen (ASC) and pepsin-soluble collagen (PSC) from swim bladders of miiuy croaker could scavenge 1,1-diphenyl-2-picrylhydrazyl (DPPH) radical, hydroxyl radical, superoxide anion radical and 2,2′-azino-bis-3-ethylbenzothiazoline-6-sulfonic acid (ABTS) radical in a dose-dependent manner and the radical scavenging activity of PSC was higher than that of ASC at all concentrations [12]. Zhuang et al. reported that jellyfish collagen (JC) and jellyfish collagen hydrolysate (JCH) alleviated UV-induced abnormal changes of antioxidant defense systems such as superoxide dismutase (SOD) and glutathione peroxidase (GSH-Px). In addition, JCH with lower molecular weight as compared to JC provides a much stronger protection against UV-induced photoaging [13]. Therefore, antioxidant collagens and collagen peptides derived from marine fish have gained enormous interest in nutraceutical, pharmaceutical and cosmeceutical industries.

Fish scales are composed of protein and collagen of connective tissue (41 to 81%) and calcium-deficient hydroxyapatite. For now, approximately 49,000 tons of fish scales are generated in the de-scaling process of aquatic products. However, large quantities of scales are discarded as waste during processing and filleting due to lower economic value, which give rise to some additional ecological environmental problems especially in developing countries. Effective use of those resources not only solves the problem of environmental pollution but also increases economic returns for the fishery industry. Therefore, collagens have been isolated from scales of some kinds of fish [14,15,16,17,18,19,20] and those results indicated that fish scale collagens are more appropriate as the alternative of pig skin collagen than fish skin collagen [21,22]. Miiuy croaker (*Miichthys miiuy*) is an important and highly consumed aquaculture species in China and Japan and it has been widely cultured since late 1990s because of its fast growth, various feeding habit and high medicinal and economic values [23]. Therefore, making full use of miiuy croaker scales to produce medical products with higher value will further accelerate the development of the miiuy croaker aquaculture industry. However, there was little information available about the extraction of collagen from the scales of miiuy croaker. In addition, there are some differences in structure and amino acid composition of collagens from different fish scales due to the living environment and species, which further influence the physicochemical and bioactive properties of collagens. Therefore, acid-soluble collagen (ASC-MC) and pepsin-soluble collagen (PSC-MC) from the scales of miiuy croaker (*M. miiuy*) were prepared and their physicochemical and antioxidant properties were characterized for their potential applications in the cosmetic and biomedical industries.

## 2. Results and Discussion

### 2.1. Proximate and Yield Analysis

Chemical compositions of scale from miiuy croaker, as well as the ASC-MC and PSC-MC derived from them are presented in Table 1. The main components of the scales were ash (47.31 g/100 g), moisture (26.37 g/100 g), protein (19.42 g/100 g) and fat (6.97 g/100 g). The high ash content (47.31 g/100 g) detected in the scales was mainly because of the calcium-deficient hydroxyappatite in the upper osseous layer and lower fibrillar plate of scales. The ash content of miiuy croaker scale was higher than those of the scales from redspot goatfish (42.31%) [23], croceine croaker (46.73%) [4] and deep-sea redfish (39.4%) [24] but lower than that of the scales from redlip croaker (48.49%) [4]. The protein content of the scales from miiuy croaker was higher than that of the scales from redlip croaker (18.47%) [4] but lower than those of the scales from redspot goatfish (34.46%) [23], croceine croaker (20.33%) [4], silver carp (37.91%) and carp (43.43%) [25]. The data indicated that the vast majority (>95%) of inorganic substances were removed from the scales of miiuy croaker by demineralization process. As shown in Table 1, ASC-MC and PSC-MC presented the similar chemical compositions, which had high content of protein (93.19 ± 1.80 and 94.87 ± 1.89 g/100 g for ASC-MC and PSC-MC, respectively) and low contents of moisture (5.18 ± 0.43 and 4.37 ± 0.32 g/100 g for ASC-MC and PSC-MC, respectively), ash (1.15 ± 0.54 and 0.92 ± 0.39 g/100 g for ASC-MC and PSC-MC, respectively) and fat (0.50 ± 0.15 and 0.34 ± 0.08 g/100 g for ASC-MC and PSC-MC, respectively). Those data indicated that the impurities in scales were effectively removed through the extraction process of collagens.

ASC-MC and PSC-MC were isolated from the scales of miiuy croaker (*M. miiuy*) with yields of 0.64 ± 0.07% and 3.87 ± 0.15% of dry weight basis, respectively. The yield of PSC-MC was 6.05-fold higher than that of ASC and it could be supposed that there were many interchain cross-links at the telopeptide region, leading to the low solubility of collagen in acid [4,8]. With further limited pepsin digestion, the cross-linked molecules at the telopeptide region were cleaved and resulted in further extraction. So, pepsin has been used to isolate collagen from the scales of redlip croakers [4], croceine croaker [15], grass carp [17], seabass [19], spotted golden goatfish [20] and snakehead [25]. Thus, pepsin could be used as an aid for increasing the extraction yield of collagen from the byproducts of miiuy croaker and other aquatic products.

### 2.2. Amino Acid Analysis

The amino acid compositions of type I collagen from calf skin (CSC), ASC-MC and PSC-MC from the scales of miiuy croaker were expressed as amino acid residues per 1000 total amino acid residues and presented in Table 2. The results indicated that ASC-MC and PSC-MC had similar amino acid compositions, with glycine (Gly) as the most abundant amino acid, followed by alanine (Ala), proline (Pro) and hydroxyproline (Hyp). Low contents of cysteine (Cys), tyrosine (Tyr), hydroxylysine (Hyl) and histidine (His) were also observed. In general, Gly represents about one-third of the total residues and is normally spaced at the beginning of typical tripeptide repetitions (Gly-X-Y, X is mostly Pro and Y is Hyp) present in areas of collagens that do not include the first 10 or so amino acids at the C-terminus and the last 14 or so amino acids at the N-terminus [3]. Moreover, Gly, as the smallest amino acid with only a hydrogen atom side chain, allows the three helical chains to form the final superhelix. In addition, Gly content of ASC-MC (341.8 residues/1000 residues) was higher than those (328–341 residues/1000 residues) of ASC from scales of Japanese seabass [26], deep-sea redfish [24], redspot goatfish [23] and common carp [27] but lower than those of scales ASC from *Labeo rohita* (361 residues/1000 residues), *Catla catla* (353 residues/1000 residues) [28], redlip croaker (351.4 residues/1000 residues) and croceine croaker (347.1 residues/1000 residues) [4]. The Gly content of PSC-MC (344.5 residues/1000 residues) was higher than those (276–350 residues/1000 residues) of PSC from scales of Japanese seabass (337 residues/1000 residues) [26], Nile tilapia (276 residues/1000 residues) [29], redspot goatfish (340 residues/1000 residues) [23] and snakehead fish (327.1 residues/1000 residues) [25] but lower than those of scales black drum 345 residues/1000 residues), sheepshead (347 residues/1000 residues) [30], *L. rohita* (361 residues/1000 residues), *C. catla* (353 residues/1000 residues) [28], bighead carp (350 residues/1000 residues) [31] and croceine croaker (347.1 residues/1000 residues) [4]. 

As shown in Table 2, the imino acid (Pro + Hyp) content of ASC-MC was 197.6 residues/1000 residues, which was analogous to those (192–204 residues/1000 residues) of ASC from scales of common carp [27], Japanese sardine [26], redspot goatfish [23] and *C. catla* [28] but significantly higher than that (160 residues/1000 residues) of ASC from deep-sea redfish scales [24]. The imino acid content of PSC-MC was 195.2 residues/1000 residues, which were similar to those (189–198.1 residues/1000 residues) of scale PSC from seabream [30], snakehead [25], redspot goatfish [23] and black drum [30] but significantly higher than that (156 residues/1000 residues) of scale PSC from bighead carp [31].

Pyrrolidine rings of imino acid enforced constraints on the conformation of the polypeptide chain and helped to strengthen the thermal stability of triple helix. It has been verified that Hyp has played an important role in stabilizing the triple-stranded helix of collagen by hydrogen bonds [6,25]. Therefore, the content of imino acid is very important for the structural integrity of collagen. So, the helices of ASC-MC and PSC-MC might be more unstable than that of CSC (216.6 residues/1000 residues) because of their low contents of imino acid.

### 2.3. SDS-PAGE and Peptide Hydrolysis Patterns of ASC-MC and PSC-MC

#### 2.3.1. SDS-PAGE Pattern of ASC-MC and PSC-MC

**Figure 1:**
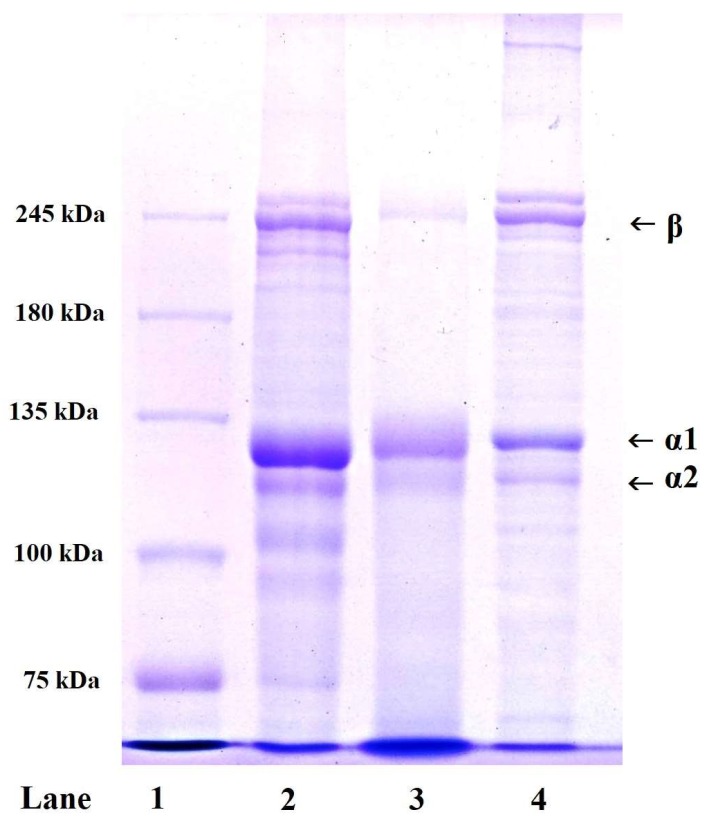
SDS-PAGE patterns of acid-soluble collagen (ASC-MC) and pepsin-soluble collagen (PSC-MC) from the scales of miiuy croaker (*M. miiuy*). Lane 1. marker protein; lane 2. ASC-MC; lane 3. PSC-MC; lane 4. type I collagen of calf skin.

SDS-PAGE pattern is commonly applied to determine the type and composition of collagen on the subunit composition, electrophoretic mobility and intensity of the band. Similar protein patterns of ASC-MC, PSC-MC and type I collagen from calf skin (CSC) were observed in Figure 1. ASC-MC and PSC-MC were composed of two different α chains (α1 and α2) with molecular weight (MW) of about 121.3 and 114.9 kDa, respectively. High molecular weight component of β (dimers) chains were also observed. Moreover, the α1-chain:α2-chain band intensity ratio of ASC-MC and PSC-MC was approximate 2:1. The result of Figure 1 including α chains (α1 and α2) and type I collagen of calf skin (Lane 4) suggested that ASC-MC and PSC-MC from the scales of miiuy croaker were mainly composed of type I collagen ([α1]_2_α2). This finding was agreement with the scale collagens from tilapia [18], snakehead fish [25], Japanese sardine [26], *L*. *rohita* and *C*. *catla* [28].

#### 2.3.2. Peptide Hydrolysis Patterns of ASC-MC and PSC-MC

**Figure 2:**
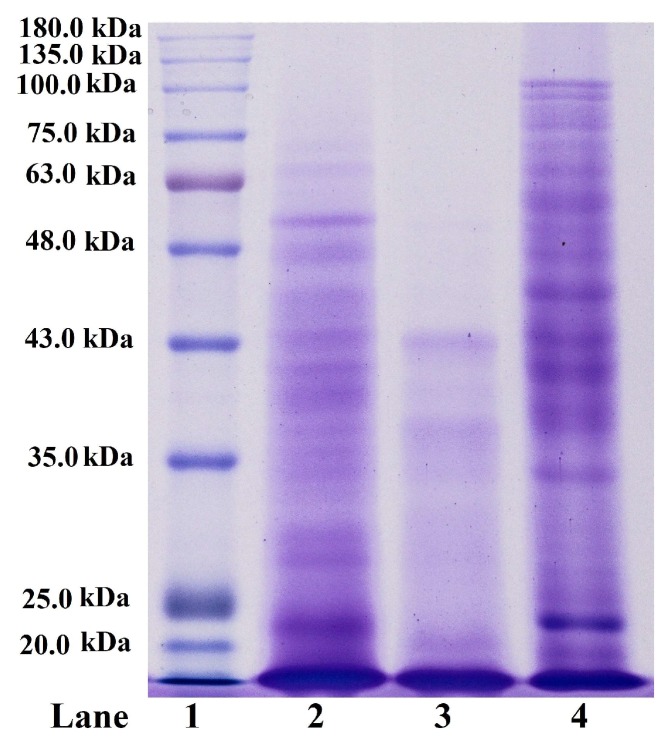
Peptide hydrolysis patterns of acid-soluble collagen (ASC-MC) and pepsin-soluble collagen (PSC-MC) from the scales of miiuy croaker (*M. miiuy*). Lane 1. marker protein; lane 2. ASC-MC; lane 3. PSC-MC; lane 4. type I collagen of pig skin (CSC).

Peptide hydrolysis patterns of ASC-MC and PSC-MC from the scales of miiuy croaker (*M. miiuy*) are presented in Figure 2. After digested by trypsin at pH of 2.5, 37 °C for 3 h, the high MW components including α-chains (α1 and α2) and β-chains almost entirely disappeared with a concomitant generation of lower MW peptide fragments ranging broadly from 20.0 to 100.0 kDa. Compared peptide hydrolysis patterns of ASC-MC and PSC-MC with that of CSC, it could be found that CSC was more tolerant to digestion by trypsin at the same conditions because the peptide fragments with high molecular weights were more than that of ASC-MC and PSC-MC, which was agreement with the analysis that the helices of ASC-MC and PSC-MC might be more unstable than that of CSC because of the lower content of imino acid. In addition, PSC-MC was easier to digestion by trypsin than ASC-MC as indicated by a greater band intensity of lower MW peptide fragments ranging from 20.0 to 43.0 kDa. Therefore, ASC-MC, PSC-MC and CSC might have some different in their primary structures and sequence amino acids, which will be our future work.

### 2.4. Ultraviolet (UV) Spectra

**Figure 3:**
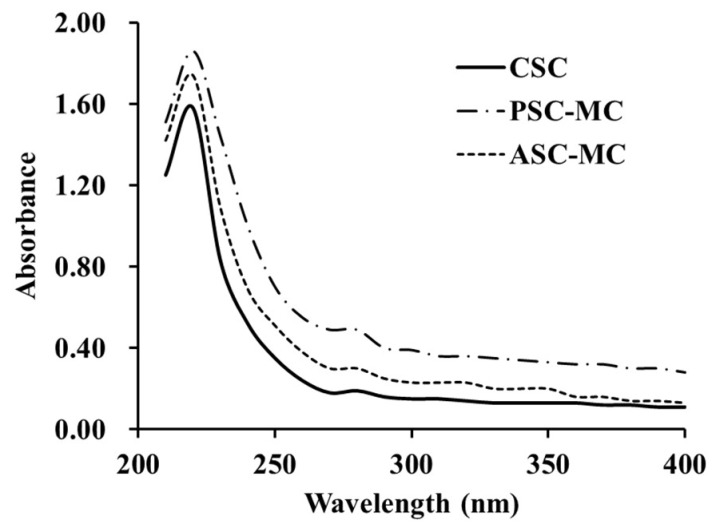
UV spectra of type I collagen from calf skin (CSC) and acid-soluble collagen (ASC-MC) and pepsin-soluble collagen (PSC-MC) from the scales of miiuy croaker (*M. miiuy*).

It is well known that the maximum absorption wavelength of protein in the near ultraviolet region is 280 nm because of the absorbance (280 nm) of aromatic amino acids such as Phe, Trp and Tyr [8]. Previous reports indicated that the protein might be collagen if there was a maximum absorption near 210–240 nm [4,22]. The UV absorption data of ASC-MC and PSC-MC were shown in Figure 3. The maximum absorption peaks of CSC, ASC-MC and PSC-MC were at 220 nm, which was related to the groups C=O, –COOH and CONH_2_ in polypeptides chains of collagens [22]. Very weak absorbance measurements were obtained at 280 nm due to low concentrations of aromatic amino acids in ASC (20.3 residues/1000 residues) and PSC (19.9 residues/1000 residues) (Table 1). Similar findings were reported in collagens from skin of loach (218 nm) [32], body wall of sea cucumber (220 nm) [33] and channel catfish (232 nm) [34].

### 2.5. Fourier-Transform Infrared Spectroscopy (FTIR)

**Figure 4:**
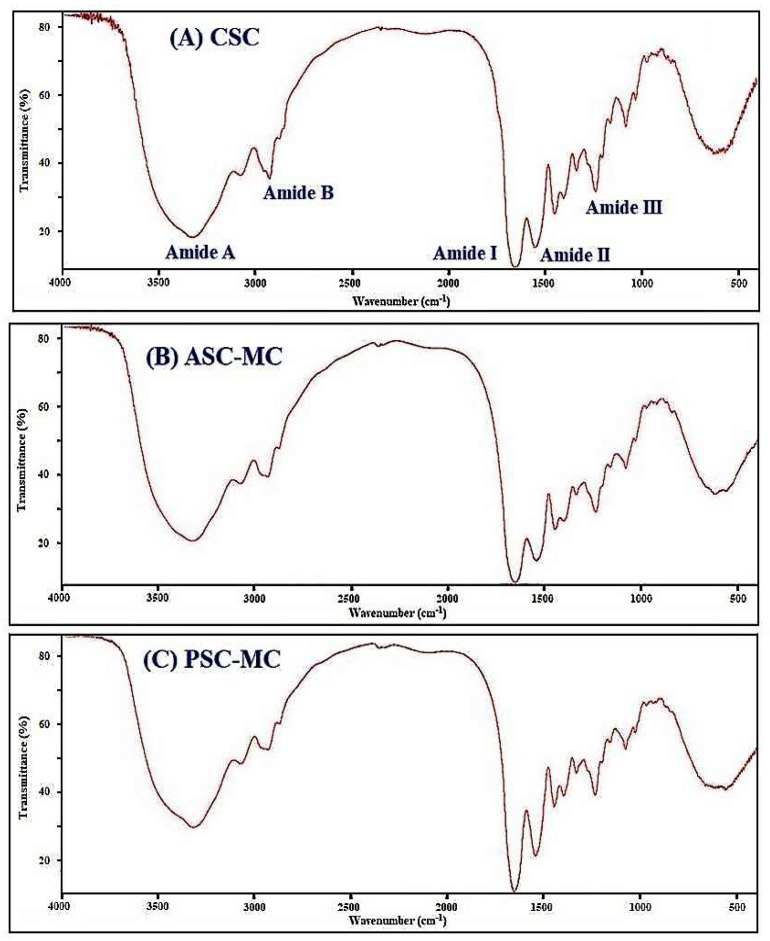
FTIR spectra of type I collagen from calf skin (CSC) (**A**) and acid-soluble collagen (ASC-MC) (**B**) and pepsin-soluble collagen (PSC-MC) (**C**) from the scales of miiuy croaker (*M. miiuy*).

FTIR spectrum is a powerful technique to research the structure and of collagens and configuration of polypeptide chain and the frequencies relate to the nature of the molecular bonds and their structure and chemical environment [35]. Collagen structure is distinguished by the formation of a right-handed triple superhelical rod consisting of three almost identical polypeptide chains. Each polypeptide chain forms a left-handed helix and consists of repeating triplets (Gly-Xaa-Yaa) [12,36]. In that collagen structure, three polypeptide strands were held together in a helical conformation by a single interstrand N-H(Gly)···O=C(Xaa) hydrogen bond per triplet [35,37]. Therefore, the characteristic peaks of amide A, B, I, II and III band contain a lot of valuable information on the right-handed triple helical conformation of collagen [4,12]. The FTIR spectra of ASC-MC, PSC-MC and CSC were shown in Figure 4 and similar FTIR spectra of ASC-MC, PSC-MC and CSC were observed. The major peaks, including amide A, amide B, amide I, amide II and amide III, could be found in amide band region and assigned in Table 3, which arise from the vibration of the peptide groups and provide information about the secondary structure of ASC-MC, PSC-MC and CSC.

The band of amide A is bound up with the N-H stretching frequency. The wavenumber of a free N-H stretching vibration is located next to the range 3400–3440 cm^−1^ and the wavenumber would move to lower frequency if the N-H group participated in the formation of a hydrogen-bond [8,38]. Figure 4 showed that the amide A wavenumbers of ASC-MC and PSC-MC were in 3415 and 3424 cm^−1^. The data illustrated that some N-H groups in ASC-MC and PSC-MC contributed to the formation of hydrogen bonding and the hydrogen-bonding numbers of ASC-MC were more than that of PSC-MC. However, the amide A wavenumbers of ASC-MC and PSC-MC were lower than that of CSC (3426 cm^−1^), which indicated that the structure stability of ASC-MC and PSC-MC was weaker than that of CSC. The amide B band is related to asymmetric stretch vibrations of ${-\mathrm{NH}}_{3}^{+}$ and =C–H and the shift of amide B to higher wavenumber is associated with an increase in free NH-${\mathrm{NH}}_{3}^{+}$ groups from lysine residues of N-terminal [8,12]. The wavenumbers of amide B band of CSC, ASC-MC and PSC-MC were found at positions of 2940, 2937 and 2936 cm^−1^, respectively. The result indicated that the free ${-\mathrm{NH}}_{3}^{+}$ groups of PSC-MC was fewer than those of ASC-MC and CSC.

Amide I, amide II and amide III bands are bound up with the triple helical structure of collagen, resulting from C=O stretching, N–H bending and C–H stretching, respectively [9,26]. The amide I band with strong absorbance in the range of 1600–1700 cm^−1^ is primarily associated with the C=O stretching vibration along the polypeptide backbone or a hydrogen bond coupled with COO– and the decrease of molecular order will make the peak shift to lower wavenumber [19]. Amide I band of ASC-MC was found at 1658 cm^−1^ and slight lower wavenumber (1655 cm^−1^) was found for PSC-MC. The result indicated that partial telopeptides were degraded by pepsin during the preparation process of PSC-MC, which caused the missing of active amino acids (Lys, Hyl and His) at telopeptide region of PSC-MC molecular [8]. 

The amide II band representing the N–H bending vibration coupled with C–N stretching vibration generally occurs in the range of 1550–1600 cm^−1^, which specifies the number of NH groups involved in hydrogen bonding with the adjacent α-chain; therefore, the lower wavenumber of the amide II band is related to the increased of hydrogen bonds by NH groups, which is attributed to collagen’s higher structure order [12]. The wavenumbers of CSC, ASC-MC and PSC-MC were found to be 1541, 1543 and 1547 cm^−1^, respectively, which indicated that the hydrogen bonding in CSC and ASC-MC was more than that of PSC-MC and the finding is consistent with the result of peptide hydrolysis patterns.

Amide III band absorption was arisen from wagging vibrations of CH_2_ groups from the Gly backbone and Pro side-chains, which is weak and associated with the triple helix structure of collagen [12]. Figure 4 showed the amide III bands of CSC, ASC-MC and PSC-MC were located at wavenumbers of 1241, 1239 and 1237 cm^−1^, respectively. The result indicated that hydrogen bonds were involved in CSC, ASC-MC and PSC-MC. In addition, the intensity ratio between Amide III band and 1450 cm^−1^ band has been used to elucidate the triplehelical structure of collagen and the absorption ratio between amide III (CSC 1241 cm^−1^, ASC-MC 1239 cm^−1^ and PSC-MC 1237 cm^−1^) and 1452 cm^−1^ (CSC), 1451 cm^−1^ (ASC-MC) or 1448 cm^−1^ (PSC-MC) bands was approximately equal to 1.0, which confirmed that ASC-MC and PSC-MC have maintained a high extent of intact triple helix structures. In addition, the amide I and amide A bands on ASC-MC and PSC-MC suggested that the structure of ASC-MC was more stable than that of PSC-MC due to more hydrogen-bonding and partial telopeptides in ASC-MC molecular but the structures of ASC-MC and PSC-MC were more unstable than that of CSC on the information of their FTIR spectra.

### 2.6. Viscosity and Denaturation Temperature (T_d_)

Collagen consists of amino acids wound together to form triple-helices to form of elongated fibrils and the triple helix structure could be depolymerized and transformed to the unordered coil configuration if the intramolecular hydrogen bond was broken by high temperature, which is along with the changes of physical characteristics, such as solubility decrease, precipitation and viscosity reducing. Therefore, viscosity measurement is often applied to research the thermos stability of collagen [4,8].

**Figure 5:**
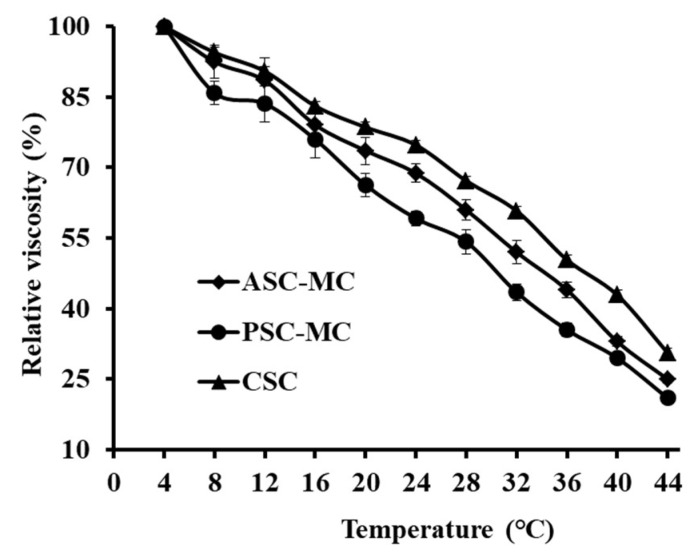
Relative viscosity changes of acid-soluble collagen (ASC-MC) and pepsin-soluble collagen (PSC-MC) from the scales of miiuy croaker (*M. miiuy*) in deionized water. All data are presented as the mean ± SD of triplicate results.

As shown in Figure 5, the relative viscosities of ASC-MC and PSC-MC solutions showed a similar rapid decline trend at the concentration of 0.6% when temperature increased from 4 to 44 °C. Denaturation temperature (*T*_d_) is the temperature at which the triple-helix structure of collagen deforms to a random coil structure. The *T*_d_ values of ASC-MC and PSC-MC were 32.2 and 29.0 °C, which were similar to those of some warm and tropical fish species, such as skipjack tuna (29.7 °C), paper nautilus (27 °C), ocellate puffer (28 °C), eel (29.3 °C), Japanese seabass (26.5 °C) and ayu (29.7 °C) [39] and higher than those of cold-water fish species, such as Alaska pollack (16.8 °C), Baltic cod (15.0 °C) and Argentine hake (10.0 °C) [40]. However, the *T*_d_ values of ASC-MC and PSC-MC were lower than those of CSC (35.9 °C). The finding further confirmed that the helix structures of ASC-MC and PSC-MC were more unstable than those of collagens from mammals. A low *T*_d_ value is an undesirable property in the manufacturing process and for biomaterials because denaturation drastically changes the biological, mechanical and physicochemical properties of collagen [41]. At present, chemical crosslinking using glutaraldehyde, carbodiimide or physical treatments including ultraviolet irradiation and dehydrothermal treatment usually was used to improve the thermal stability of collagen from aquatic animals [41]. The *T*_d_ and viscosity of PSC-MC were slightly lower than this of ASC-MC, which might be caused by MW reduction in the telopeptide region induced by pepsin hydrolysis.

### 2.7. Solubility

**Figure 6:**
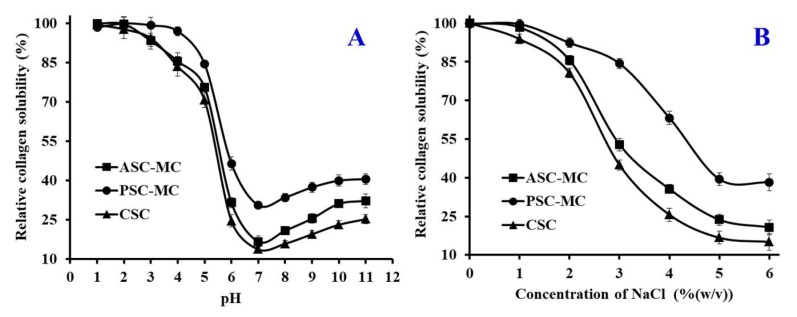
Solubilities of acid-soluble collagen (ASC-MC) and pepsin-soluble collagen (PSC-MC) from the scales of miiuy croaker (*M. miiuy*) in 0.5 M acetic acid at different pH (**A**) and NaCl concentrations (**B**). All data are presented as the mean ± SD of triplicate results.

Solubility of collagen is the most important factor and excellent index for their functionality. Knowledge of collagen solubility can give useful information on the potential utilization of proteins and their functionality, especially in foams, emulsions and gels [42]. In addition, solubility is the main characteristic of collagens selected for use in liquid foods and beverages. Except influenced by amino acid composition and sequence, molecular weight and conformation, solubility of collagen is affected by environmental factors, such as pH, ionic strength, type of solvent, temperature and processing conditions [43]. Therefore, the influence of pH and ionic strength (NaCl concentration) on solubility of ASC-MC and PSC-MC was measured and the results were shown in Figure 6.

#### 2.7.1. Effect of pH

Collagen to be soluble should be able to interact as much as possible with the solvent. Collagen-water interactions increase at pH values higher or lower than the isoelectric point (p*I*) because Collagen carries a positive or negative charge [43]. However, collagens have a net zero charge at the p*I*, attractive forces predominate and molecules tend to associate, resulting in insolubility. Figure 6A depicted that ASC-MC, PSC-MC and CSC were more easily dissolved in acid solution (pH 1–5) and the solubility significantly decreased at pH 5–7. The maximum solubility of ASC-MC was achieved at pH 1 and the solubility of PSC-MC reached maxima at pH 1–3. Similar result was reported for scale collagens from redspot goatfish [23], snakehead [25], *C. catla* [28], croceine and redlip croakers [4]. The minimum solubility of ASC-MC and PSC-MC was at pH 7. However, the solubility of ASC-MC and PSC-MC showed a slight upward trend when pH value was higher than 7. The present data indicated that the p*I*s of ASC-MC and PSC-MC were about pH 7 and the data were in agreement with previous reports that the collagen p*I*s were usually at pH 6–9 [23]. At the same pH value, PSC-MC had higher solubility than ASC-MC, which was in line with that of PSC from scales of redspot goatfish [23], croceine and redlip croakers [4]. The finding could be due to the predominance of weaker bonds and lower cross-linking degree of PSC.

#### 2.7.2. Effect of NaCl Concentration

The functionality of collagens can be studied more effectively if a systematic study is first made of the protein solubility under various ionic conditions [43]. The mechanism of the ionic strength effect on protein solubility probably involves solvation, electrostatic and salting in and salting out phenomena [8]. Low concentrations of neutral salts may increase the solubility of proteins. Chloride ions increase solubility by electrostatic repulsion after binding to the positively charged protein groups. As presented in Figure 6B, the solubility of ASC-MC, PSC-MC and CSC showed similar pattern with slightly difference when NaCl concentrations ranged from 0 to 6%. The solubility of ASC-MC, PSC-MC and CSC remained high level (more than 90%) when the NaCl concentration was lower than 1% and rapidly reduced if NaCl concentration was between 1 and 5%, after which the solubility of ASC-MC, PSC-MC and CSC was slowly reduced when the NaCl concentration was ranged from 5 to 6%. The result was like the solubility of scale collagens from redspot goatfish [23], snakehead [25], bighead carp [27], croceine and redlip croakers [4]. The solubility changes of ASC-MC, PSC-MC and CSC might be due to the ‘salting out’ effect resulted from the relatively high NaCl concentration. An ionic strength increase could enhance the hydrophobic-hydrophobic interactions of protein chains and increase the competition for water with the ionic salts, which led to protein precipitation [4]. These solubility behaviors of ASC-MC, PSC-MC and CSC with pH and NaCl concentration changes might play an important role in their preparation process.

### 2.8. Zeta Potential

**Figure 7:**
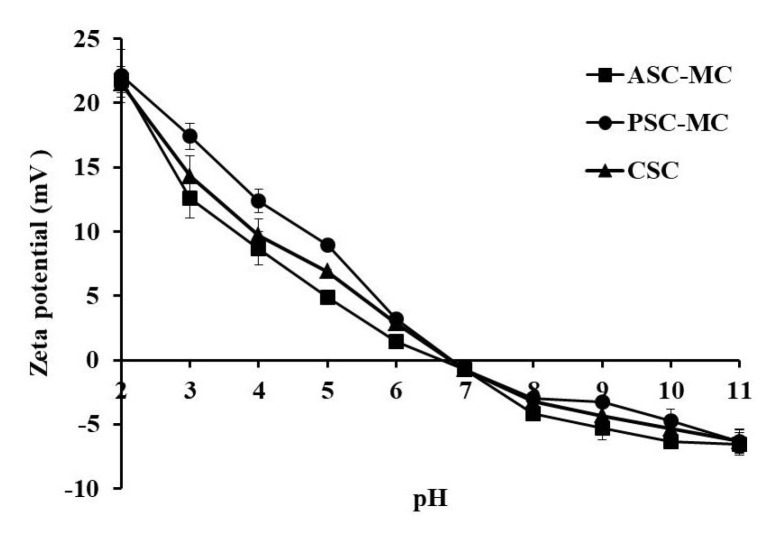
Zeta potentials of acid-soluble collagen (ASC-MC) and pepsin-soluble collagen (PSC-MC) from the scales of miiuy croaker (*M. miiuy*) at different pH levels. All values were mean ± SD.

Zeta potential, also known as electrokinetic potential, is the potential difference across phase boundaries between solids and liquids and often used to describe double-layer properties of a colloidal dispersion [12]. Therefore, the zeta potential is a key indicator of the stability of colloidal dispersions and macromolecules with a high zeta potential have low propensity to form aggregates [44]. The zeta potentials of the ASC-MC, PSC-MC and CSC at various pH values were presented in Figure 7 and showed the similar tendency. ASC-MC and PSC-MC were positively charged at pH 2–6 and negatively charged at pH 7–11. The Zeta potential data revealed when the zeta net charges of ASC-MC and PSC-MC were zero, their potential values and p*I* values were 6.66 and 6.81, respectively, which consistent with the result obtained in effect of pH on solubility that the p*I*s of ASC-MC and PSC-MC were about pH 7. The difference in p*I* values between ASC-MC and PSC-MC might be due to the removal of PSC telopeptides by pepsin. Collagen from various fish skins had different p*I* values, such as ASC from scales and skin of tilapia (6.82 and 6.42 respectively) [29], ASC and PSC from skin of loach (6.42 and 6.51 respectively) [32] and PSC from skin of bamboo shark (6.12) [45]. The differences in collagen p*I* values might due to amino acid sequences and distribution of amino acid residues.

### 2.9. Collagen Ultrastructure

**Figure 8:**
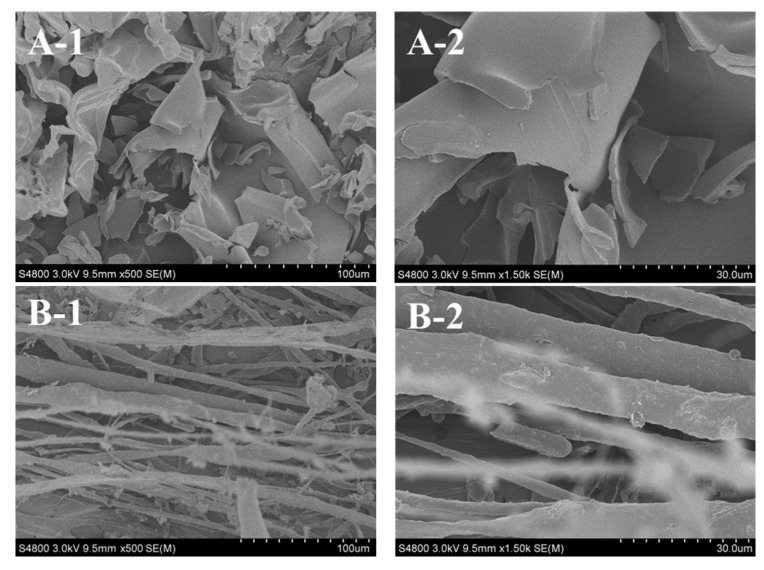
SEM images of acid-soluble collagen (ASC-MC) and pepsin-soluble collagen (PSC-MC) from the scales of miiuy croaker (*M. miiuy*). (**A**): ASC-MC; (**B**): PSC-MC. 1: (×500); 2: (×1500).

Ultrastructure and surface area of collagen are important to evaluate its potential applications in biomedicine and biomedicine engineering [23,30]. Scanning electron microscopy (SEM) ultrastructure of ASC-MC and PSC-MC from the scales of miiuy croaker were observed in Figure 8. ASC-MC presented irregular dense sheet-like film linked by random-coiled filaments under SEM (Figure 8A) and the surface was partially wrinkled, possibly because of dehydration during lyophilizing. The fibrillar structure of PSC-MC was also found in Figure 8B. SEM ultrastructure of ASC-MC and PSC-MC was similar to those of collagens from skin and bone of Spanish mackerel [15], gutted silver carp [30], swim bladder of carp [23] and skin of Amur sturgeon [46]. In addition, the sheet-like film structure of ASC-MC and the fibrillar structure of PSC-MC at the same concentration (5% (*w*/*v*)) suggested that there were some differences in the primary structures between ASC-MC and PSC-MC. Previous reports suggested that collagens with interconnectivity, fibrillary and sheet-like film structures have the potentiality to be used in new tissue formation, cell seeding, growth, wound healing and mass transport and migration [30]. In general, the microscopic structure of ASC-MC and PSC-MC indicated that they may be the suitable biomaterial for different medical applications.

### 2.10. Antioxidant Activity

Oxidative stress is associated with the pathogenesis of many chronic diseases and excessive free radicals generated in metabolism are a potential reason for the oxidative stress of human body [11,47]. Reactive oxygen species (ROS), such as superoxide and hydroxyl radical, are observed to possess the strong capacity to attack biological macromolecules, which further cause cell injury and play an important role in developing process of chronic diseases [3,48]. In addition, the radical scavenging activity is an important property for skin photoaging and ultraviolet damage of cosmeceutical products [49,50]. Therefore, the radical scavenging activity of the fish collagen is an important characteristic for evaluating its potential application. 

**Figure 9:**
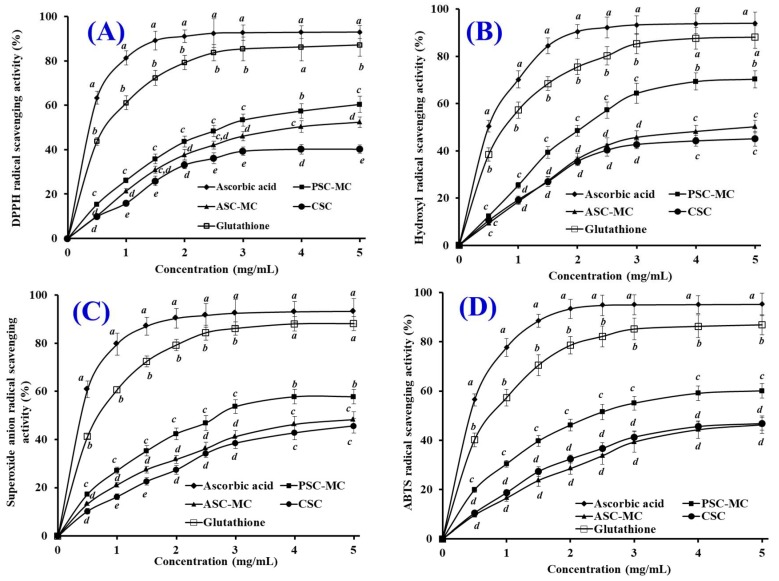
DPPH radical (**A**), hydroxyl radical (**B**), superoxide anion radical (**C**) and ABTS radical (**D**) scavenging activities of CSC and acid-soluble collagen (ASC-MC) and pepsin-soluble collagen (PSC-MC) from the scales of miiuy croaker (*M. miiuy*). Ascorbic acid and glutathione were designed as positive controls to compare with the sample groups. All the values were mean ± SD. ^a–e^ Values with same letters indicated no significant difference of different sample at same concentrations (*p* > 0.05).

The antioxidant properties of ASC-MC and PSC-MC were evaluated using DPPH radical, hydroxyl radical, superoxide anion radical and ABTS radical scavenging assays and shown in Figure 9. The present results indicated that the antioxidant capacities (DPPH radical, hydroxyl radical, superoxide anion radical and ABTS radical scavenging activities) of ASC-MC and PSC-MC were positively correlated with their tested concentration ranged from 0 to 5 mg/mL. The DPPH radical scavenging activity of PSC-MC was significantly higher than those of ASC-MC and CSC at the same concentrations except the concentrations of 1.5 and 2.5 mg/mL (*p* < 0.05) and the hydroxyl radical scavenging activity of PSC-MC showed the same trend and was significantly higher than those of ASC-MC and CSC at the same concentrations except the concentrations of 0.5 mg/mL (*p* < 0.05). Furthermore, PSC-MC showed significantly higher superoxide anion radical and ABTS radical scavenging activity than PSC-MC did at the same concentrations (*p* < 0.05). However, there were no significant difference between ASC-MC and CSC on hydroxyl radical, superoxide anion radical and ABTS radical scavenging activities at most tested concentrations (*p* > 0.05). In addition, the radical scavenging activities of ASC-MC, PSC-MC and CSC were significantly lower than those of the positive control of ascorbic acid and glutathione (GSH), which was agreed with the previous reports that small molecular including oligopeptides (2–9 amino acid residues) showed high radical scavenging activities than macromolecules because they were easily accessible to active radicals to provide potential effects in reaction mixture [51,52]. 

Moreover, the scavenging capability of PSC-MC on hydroxyl radical (Figure 9B) and superoxide anion radical (Figure 9C) was higher than those of DPPH radical and ABTS radical. In the human body, superoxide anion radical is the most common free radical generated in vivo. It can produce hydrogen peroxide and hydroxyl radical through dismutation and other types of reactions in vivo. Both superoxide anion radical and its derivatives including hydroxyl radical are cell damaging, which can cause damage to DNA and membrane of cell [15]. Therefore, the damage caused by the highly reactive free radicals is widely accepted as the primary reason for skin damage, inflammation and skin aging [11]. In biological systems, SODs can catalyze superoxide radicals into hydrogen peroxide and oxygen with a reaction rate 10,000-fold higher than that of spontaneous dismutation. Therefore, ASC-MC and PSC-MC might have a high antioxidant activity similar to that of SOD and could be served as hydroxyl radical and superoxide radical scavenger in cosmeceutical products for reducing the radical damage in skin aging.

The skin aging process can be divided into intrinsic aging and photoaging. Skin photoaging is a premature skin-aging damage after repeated exposure to ultraviolet (UV) radiation, mainly characterized by oxidative stress and inflammatory disequilibrium, which makes skin show the typical symptoms of photoaging such as coarse wrinkling, dryness, irregular pigmentation and laxity [53]. ROS are thought to be involved in cancer, aging and various inflammatory disorders. Therefore, more and more attention has been paid to utilize fish-derived collagen, gelatin and peptides for protecting skin from photoaging due to their excellent antioxidant activity and skin-repairing ability [12]. Chen et al. reported that gelatin hydrolysate (CH) with average molecular weight of 1200 Da from pacific cod skin can improved pathological changes of collagen fibers and significantly inhibited collagen content reduction in photoaging skin. Moreover, CH can effectively protect against UV irradiation-induced skin photoaging by inhibiting the expression and the activity of matrix metalloproteinases [54,55]. Wang et al. reported that the collagen polypeptides from *Apostichopus japonicus* showed protective effects against ultraviolet radiation-induced skin photoaging [56]. Hou et al. reported collagen polypeptide fractions of CP1 (2 kDa < MW < 6 kDa) and CP2 (MW < 2 kDa) from cod skin could protect skin structures against UV-induced wrinkle formation and destruction and they also provided good moisture absorption and retention properties [57]. Sun et al. reported that tilapia gelatin peptides (TGP) could protect skin lipid and collagen from the UV radiation damages through alleviating the UV-induced abnormal changes of antioxidant indicators and repairing the endogenous collagen synthesis [1]. Therefore, the present finding suggested that ASC-SC and PSC-SC from the scales of miiuy croaker might be served as hydroxyl radical and superoxide anion radical scavenger in cosmeceutical products for protecting skins from photoaging and ultraviolet damage. 

## 3. Experimental Section

### 3.1. Chemicals and Reagents

The scales of miiuy croaker (*M*. *miiuy*) were obtained from Zhejiang Hailisheng Group Co. Ltd., in Zhoushan City, Zhejiang Province of China. High molecular weight markers and type I collagen from calf skin (CSC) were used as the standards and bought from Sigma-Aldrich (St. Louis, MO, USA). All other reagents used were of analytical grade.

### 3.2. Extraction of Scale Collagens

#### 3.2.1. Pretreatment of Scales

The extraction procedure of scale collagens of miiuy croaker was according to the method described by Matmaroh et al. [20]. The scales were added to 0.1 M NaOH solution with a material/liquid ratio of 1:10 (*w*/*v*) and stirred for 6 h and NaOH solution was replaced each 3 h. Afterwards, the scales were rinsed using cold tap water until the pH value of washing water got to 7.0–7.5 and demineralized using EDTA-2Na (0.5 M, pH 7.4) with a material/liquid ratio of 1:10 (*w*/*v*) and stirred for 48 h and EDTA-2Na solution was renewed every 12 h. The pretreated scales were cleaned with a scale/cold tap water ratio of 1:20 (*w*/*v*) for three times.

#### 3.2.2. Extraction of Acid-Soluble Collagen (ASC-MC)

The pretreated scales were soaked in 0.5 M acetic acid solution with a material/liquid ratio of 1:15 (*w*/*v*) for 48 h. The extracting solution was filtered using a cheesecloth and the collagen was precipitated from the filtrate using 2.5 M NaCl solution. The precipitates were collected by centrifugation at 20,000× *g* for 30 min at 4 °C, re-dissolved in a minimum volume of 0.5 M acetic acid solution and dialyzed against 25 volumes of 0.1 M acetic acid solution for 12 h. Thereafter, the dialyzed solution was dialyzed against 25 volumes of distilled water for 48 h and distilled water was changed each 12 h. The final dialysate was lyophilized.

#### 3.2.3. Extraction of Pepsin-Soluble Collagen (PSC-MC)

The scale residues from ASC-MC preparation were soaked in 0.5 M acetic acid solution containing 1% porcine pepsin (*w*/*w*) at a solvent/scale ratio of 15:1 (*v*/*w*) for 48 h at 4 °C. Thereafter, other processes were carried with the identical manner as the ASC-MC preparation.

### 3.3. Proximate Analysis

Moisture, ash and fat contents of scale and collagens were determined using the methods of Association of Official Agricultural Chemists (AOAC) method (2003) with the method numbers of 950.46B, 920.153 and 960.39 (a), respectively. Protein content was measured using the Kjeldahl method and an auto protein analyzer (Kjeltec 2400 auto-analyzer, Hillerød, Denmark). The converting factor of 6.25 was used for calculation of protein content [7].

### 3.4. Amino Acid Analysis

Tested samples were hydrolyzed in 6 M HCl at 110 °C for 24 h and the hydrolysates were vaporized and the residues were dissolved in 25 mL citric acid buffer solution. An aliquot of 0.05 mL was applied to an automated amino acid analyzer (HITACHI 835-50 Amino Acid Analyzer, Tokyo,, Japan). Then the degrees of Pro and Lys hydroxylation (%) were calculated as follows:

Degrees of Pro hydroxylation (%) = Hyp content/(Hyp content + Pro content) × 100%.

Degrees of Lys hydroxylation (%) = Hyl content/(Hyl content + Lys content) × 100%.

### 3.5. Electrophoretic Pattern

Electrophoretic patterns of ASC-MC and PSC-MC were determined using the previous method [9], using 7.5% resolving gel and 4% stacking gel. Collagen samples were suspended in 5% (*w*/*v*) SDS prior to incubation at 85 °C for 1 h. The mixture was centrifuged at 5,000*× g* for 10 min for removing undissolved debris. The samples (about 20 μL) were mixed with the sample loading buffer (60 mM Tris-HCl, pH 8.0, containing 25% glycerol, 2% SDS, 0.1% bromophenol blue) at the ratio of 4:1 (*v*/*v*) in the presence of β-ME, then applied to sample wells and electrophoresed in an electrophoresis instrument (AE-6200, ATTO Corporation, Tokyo, Japan). The electrophoresis was carried out for about 4 h at a constant voltage of 100 V. After electrophoresis, gel was stained with 0.1% (*w*/*v*) Coomassie blue R-250 in 45% (*v*/*v*) methanol and 10% (*v*/*v*) acetic acid.

### 3.6. Peptide Hydrolysis Patterns

Peptide hydrolysis patterns of ASC-MC and PSC-MC were measured on the method described by Wu et al. [4]. Collagen solutions (3.5 M) dissolved in acetic acid solution (0.5 M) were hydrolyzed for 3.0 h at 37 °C after adding trypsin with a substrate/enzyme ratio of 20:1 (*w*/*w*) and the hydrolysis was terminated in boiled water for 5 min after the addition of SDS-PAGE sample buffer. SDS-PAGE with 12.0% separating gels was used to measure the molecular weight of peptides.

### 3.7. UV Measurements

The UV adsorption spectra of ASC-MC and PSC-MC were recorded using the method of Yu et al. [22], using a spectrophotometer (UV-1800, Mapada Instruments Co., Ltd., Shanghai, China) from 200 to 400 nm. The sample was prepared by dissolving the collagen in 0.5 M acetic acid solution with a sample/solution ratio of 1:1 000 (*w*/*v*).

### 3.8. FTIR Spectral Analysis 

The IR spectra of ASC-MC and PSC-MC were recorded in KBr disks with a FTIR spectrophotometer (Nicolet 6700, Thermo Fisher Scientific Inc., Waltham, MA, USA). The mixture at a sample to potassium bromide (KBr) ratio of 1:100 (*w*/*w*) was pressed into a disk for spectrum recording. The IR spectra in the range of 4000–400 cm^−1^ with automatic signal gain were collected in 32 scans at a resolution of 4 cm^−1^ and were ratioed against a background spectrum recorded from the clean empty cell.

### 3.9. Viscosity

Viscosity of ASC-MC and PSC-MC was measured using the previous method [9]. All the samples were dissolved in deionized water with the vibration of THZ-100 shaker (Shanghai Yiheng Technical Co., Ltd., Shanghai, China), to obtain a concentration of 0.6% (*w*/*v*) and 500 mL solutions were subjected to viscosity measurement using a NDJ-8S viscometer (Jingtian Instruments Co., Ltd., Shanghai, China) with appropriate spindles (from No.4 to No.1) and an appropriate speed. All the sample solutions were heated from 4 to 44 °C with a heating rate of 4 °C/min and the solution was held for 30 min prior to viscosity determination at the designated temperature. The relative viscosity was calculated in comparison with that obtained at 4 °C and T_d_ was defined as the temperature at which relative viscosity was 0.5.

### 3.10. Solubility

Effects of pH and NaCl concentration on the collagen solubility were measured using the previous method [9]. Collagen solutions (3.5 M) were prepared using 0.5 M acetic acid solution and stirred for 24 h at 4 °C. The solutions were centrifuged at 10,000× *g* for 15 min at 4 °C and the resulting supernatants were used for measuring solubility of collagen.

#### 3.10.1. Effect of pH on Solubility

Sample solution (8 mL) was transferred to a 50 mL centrifuge tube and the pH was adjusted with either 6 M NaOH or 6 M HCl to obtain the final pH ranging from 1 to 11. The volume of solution was made up to 10 ml by deionized water previously adjusted to the same pH as the sample solution. The solution was centrifuged at 15,000× *g* for 60 min at 4 °C. For all the samples, protein content in the supernatant was measured. Then the relative solubility was calculated in the comparison with that of be obtained at the pH giving the highest solubility. 

#### 3.10.2. Effect of NaCl on Solubility

Sample solution (5 mL) was mixed with 5 mL of NaCl in 0.5 M acetic acid at various concentrations to give the final concentrations of 0%, 1%, 2%, 3%, 4%, 5% and 6%. The mixture was stirred continuously at 4 °C for 30 min, followed by centrifuging at 15,000× *g* for 60 min at 4 °C.

### 3.11. Zeta Potential

Zeta potentials of ASC-MC and PSC-MC were measured on the previous method [29]. ASC-MC and PSC-MC were dissolved in 0.05 M acetic acid to a final con-centration of 0.2 mg/mL and incubated at 4 °C for 48 h. The zeta potentials of ASC-MC and PSC-MC were determined using a NanoBrook Omni zeta potential analyzer (Brookhaven Instruments Corporation, Holtsville, NY, USA) as reported by Chen et al. [29]. The pH of the samples (20 mL) was adjusted across a pH range (3–11) with 1 M KOH and 1 M HCl. The p*I*s of ASC-MC and PSC-MC were determined from the pH value that resulted in a zero zeta potential.

### 3.12. Collagen Ultrastructure

The morphological characteristics of ASC-MC and PSC-MC were studied by SEM using Hitachi TM-1000 (Tokyo, Japan). Collagen was re-dissolved in 0.5 M acetic acid at a concentration of 5% (*w*/*v*), followed by dialyzing against distilled water. The collagen was lyophilized in a freeze dryer (EYELA FD-1000, Tokyo Rikakikai Co., LTD, Tokyo, Japan) and the sample was sputter coated for 90 s with gold using a JEOL JFC-1200 (Tokyo Rikakikai Co., Ltd., Tokyo, Japan) fine coater. The morphologies of the electro spun fibers and membrane were observed using Hitachi TM-1000 (Hitachi High-Technologies Co., Ltd., Tokyo, Japan).

### 3.13. Antioxidant Activity

The radical (DPPH radical, hydroxyl radical, superoxide anion radical and ABTS radical) scavenging activity and lipid peroxidation inhibition assays were performed according to previously reported methods [58,59].

### 3.14. Statistical Analysis

All experiments were carried out in triplicate. An ANOVA test using the software of SPSS 19.0 (Statistical Program for Social Sciences, SPSS Corporation, Chicago, IL, USA) as applied to compare the average values of each treatment. Duncan’s multiple range test (*p* < 0.05) was used to measure the significant differences between the parameters means.

## 4. Conclusions

In the experiment, acid-soluble collagen (ASC-MC) and pepsin-soluble collagen (PSC-MC) from the scales of miiuy croaker (*M. miiuy*) were isolated and characterized. Amino acid composition, SDS-PAGE pattern, UV spectra and FTIR confirmed that ASC-MC and PSC-MC were mainly composed of type I collagen. The antioxidant capacities of ASC-MC and PSC-MC were positively correlated with their tested concentration ranged from 0 to 5.0 mg/mL and the radical scavenging activity of PSC-MC was significantly higher than that of ASC-MC at most tested concentrations (*p* < 0.05). The present result suggested that ASC-SC and PSC-SC from the scales of miiuy croaker could be served as substitutes of skin collagens from mammalian and aquatic products in cosmeceutical products for protecting skins from photoaging and ultraviolet damage by scavenging reactive oxide species. Therefore, this study provides scientific basis for the medical application of scale collagens of miiuy croaker (*M. miiuy*).

## Figures and Tables

**Table 1:** Chemical compositions of miiuy croaker scales, acid-soluble collagen (ASC-MC) and pepsin-soluble collagen (PSC-MC) from the scales of miiuy croaker (*M. miiuy*).

Sample	Proximate Compositions (g/100 g Dry Weight)				Yield (%)
	Moisture	Fat	Ash	Protein	Dry Weight Basis
Scales	26.37 ± 0.18 ^a^	6.94 ± 0.43 ^a^	47.31 ± 3.07 ^a^	19.42 ± 0.86 ^a^	
ASC-MC	5.18 ± 0.43 ^b^	0.50 ± 0.15 ^b^	1.15 ± 0.54 ^b^	93.19 ± 1.80 ^b^	0.64 ± 0.07 ^a^
PSC-MC	4.37 ± 0.32 ^b^	0.34 ± 0.08 ^b^	0.92 ± 0.39 ^b^	94.87 ± 1.89 ^b^	3.87 ± 0.15 ^b^

All values are mean ± SD (*n* = 3); ^a–b^ Values with different letters in the same column indicate significant difference (*p* < 0.05).

**Table 2:** Amino acid composition of type I collagen from calf skin (CSC), acid-soluble collagen (ASC-MC) and pepsin-soluble collagen (PSC-MC) from the scales of miiuy croaker (*M. miiuy*) (residues/1000 residues).

Amino Acid	ASC-MC	PSC-MC	CSC
Hydroxyproline (Hyp)	85.6 ± 3.3	84.8 ± 2.5	95.1 ± 2.4
Aspartic acid/asparagine (Asp)	39.6 ± 1.5	41.2 ± 1.7	45.7 ± 2.1
Threonine (Thr)	25.7 ± 1.1	27.1 ± 1.0	18.4 ± 0.8
Serine (Ser)	31.4 ± 1.3	25.5 ± 1.1	33.2 ± 0.9
Glutamine/glutamic acid (Glu)	61.9 ± 2.2	63.3 ± 3.5	75.9 ± 3.3
Proline (Pro)	112.0 ± 1.9	110.4 ± 2.9	121.5 ± 3.4
Glycine (Gly)	341.8 ± 4.2	344.5 ± 3.2	330.6 ± 4.6
Alanine (Ala)	122.3 ± 3.7	120.1 ± 3.5	119.7 ± 2.7
Cysteine (Cys)	2.3 ± 0.1	3.1 ± 0.1	0.0
Valine (Val)	22.4 ± 0.5	23.6 ± 0.7	21.5 ± 0.7
Methionine (Met)	14.3 ± 0.4	13.9 ± 0.6	6.1 ± 0.3
Isoleucine (Ile)	12.7 ± 0.5	11.5 ± 0.6	11.4 ± 0.5
Leucine (Leu)	22.7 ± 0.8	24.6 ± 0.9	23.4 ± 0.4
Tyrosine (Tyr)	5.9 ± 0.3	4.6 ± 0.3	3.7 ± 0.5
Phenylalanine (Phe)	14.4 ± 0.9	15.3 ± 1.1	3.3 ± 0.6
Hydroxylysine (Hyl)	6.2 ± 0.3	6.6 ± 0.4	7.7 ± 0.4
Lysine (Lys)	25.5 ± 1.0	24.8 ± 0.8	26.5 ± 1.1
Histidine (His)	7.6 ± 0.3	8.5 ± 0.5	5.3 ± 0.3
Arginine (Arg)	45.7 ± 1.5	46.6 ± 1.3	51.0 ± 1.4
Total	1000.0	1000.0	1000.0
Imino acid (Pro + Hyp)	197.6	195.2	216.6

All data are presented as the mean ± SD of triplicate results.

**Table 3:** FTIR spectra peak locations of CSC (type I collagen from calf skin), acid-soluble collagen (ASC-MC) and pepsin-soluble collagen (PSC-MC) from the scales of miiuy croaker (*M. miiuy*).

Properties	Peak Wavenumber (cm^−1^)			Assignment
	ASC-MC	PSC-MC	CSC	
Amide A	3415	3424	3426	NH stretch coupled with hydrogen bond
Amide B	2937	2936	2940	CH_2_ asymmetrical stretch
Amide I	1658	1655	1660	C=O stretch/hydrogen bond coupled with COO–
Amide II	1543	1547	1541	NH bend coupled with CN stretch
Amide III	1239	1237	1241	NH bend coupled with CN stretch
